# Biodegradable Gene Carriers Containing Rigid Aromatic Linkage with Enhanced DNA Binding and Cell Uptake

**DOI:** 10.3390/polym10101080

**Published:** 2018-09-29

**Authors:** Ju-Hui Zhang, Hui-Zhen Yang, Ji Zhang, Yan-Hong Liu, Xi He, Ya-Ping Xiao, Xiao-Qi Yu

**Affiliations:** Key Laboratory of Green Chemistry and Technology (Ministry of Education), College of Chemistry, Sichuan University, Chengdu 610064, China; jhzhang429@163.com (J.-H.Z.); 15737704325@163.com (H.-Z.Y.); yanhongliu@scu.edu.cn (Y.-H.L.); seacyhe@163.com (X.H.); 15982103680@163.com (Y.-P.X.)

**Keywords:** non-viral vectors, gene delivery, polyethylenimine, structure–activity relationship

## Abstract

The linking and modification of low molecular weight cationic polymers (oligomers) has become an attracted strategy to construct non-viral gene carriers with good transfection efficiency and much reduced cytotoxicity. In this study, PEI 600 Da was linked by biodegradable bridges containing rigid aromatic rings. The introduction of aromatic rings enhanced the DNA-binding ability of the target polymers and also improved the stability of the formed polymer/DNA complexes. The biodegradable property and resulted DNA release were verified by enzyme stimulated gel electrophoresis experiment. These materials have lower molecular weights compared to PEI 25 kDa, but exhibited higher transfection efficiency, especially in the presence of serum. Flow cytometry and confocal laser scanning microscopy results indicate that the polymers with aromatic rings could induce higher cellular uptake. This strategy for the construction of non-viral gene vectors may be applied as an efficient and promising method for gene delivery.

## 1. Introduction

In recent decades, gene therapy has been regarded as a promising strategy to cure multiple inherited and infectious diseases [1,2,3,4,5]. Due to the numerous obstacles during gene delivery—such as gene packing, cellular uptake, endosomal escapem and translocation into the nucleus [6]—successful gene transfection requires appropriate gene vectors. Compared to viral vectors, cationic polymers exhibit several advantages including inherent low immunogenicity, convenient production, as well as tunable surface and structure [7,8,9]. Various of polycation carriers like poly-l-lysine (PLL), polyethylenimine (PEI), poly(2-(dimethylamino) ethyl methacrylate) (PDMAEMA), and polyamidoamine (PAMAM) dendrimers have been explored [8,10,11,12]. However, the high cytotoxicity came from their polycationic characters and relatively low transfection efficiency when compared to viral vectors largely limited their clinical application. Therefore, designing safe and efficient non-viral delivery vehicles is still a pivotal challenge for gene therapy.

Among the cationic polymers, PEI is one of the most widely studied gene vectors owing to the good DNA condensation ability and successful endosomal escape ability by the “proton sponge effect” [13,14]. Initial research has proven that the transfection efficiency of PEI is molecular weight-dependent. Usually, high molecular weight (HMW) PEI has excellent transfection ability, but it is also accompanied with significant cytotoxicity. On the contrary, low molecular weight (LMW) PEI is nontoxic but exhibits low transfection efficiency [15,16,17]. To solve this conflict, more and more attention has been paid to the modification of LMW PEI [18,19,20,21,22,23,24].

One prerequisite for the gene vector based on LMW PEI is effective DNA condensation ability to avoid low transfection efficiency caused by colloidal instability and premature release of DNA [25]. Although a number of strategies to enhance interaction with DNA have been reported, their gene transfection efficiencies remained low, most probably because the enhanced DNA binding ability prevented the efficient release of the entrapped DNA [26,27,28]. Therefore, an ideal gene carrier requires a fine balance between condensation and release. Our previous studies have found that rigid aromatic moieties would benefit the interaction between the polymeric materials and DNA through the intercalation of aromatic ring into the DNA base pairs [29,30]. On the other hand, it has been proven that the β-amino esters formed by Michael addition could undergo hydrolysis or enzymatic cleavage in the cytoplasm. This biodegradable behavior endows such materials with excellent DNA release capacity and good biocompatibility [31,32,33,34,35].

Herein, we attempt to introduce rigid and aromatic rings in the polymer backbone with the aim to improve the DNA affinity. Meanwhile, ester bond that have been widely reported with biodegradable properties are used to regulate the release of DNA and balance cytotoxicity. The results of in vitro gene transfection demonstrated that although these polymers have much lower molecular weight than PEI 25 kDa, they achieved comparable or superior transfection efficiency compared to this “golden standard”. In addition, several experiments confirmed that larger aromatic rings are beneficial for DNA binding and cellular uptake. Moreover, their biodegradable properties were also verified by enzyme stimulated gel electrophoresis assays.

## 2. Experimental Section

### 2.1. Materials and Methods

All of the common chemicals and reagents were obtained commercially and were used as received. Anhydrous methanol and dichloromethane were dried and purified under nitrogen by using standard methods and were distilled immediately before use. LMW-PEI (branched, average *M*_w_ = 600 Da) was purchased from Aladdin (Shanghai, China). HMW-PEI (branched, average *M*_w_ = 25 kDa) and elastase from porcine pancreas was purchased from Sigma-Aldrich (St. Louis, MO, USA). The plasmids used in the study were pGL-3 (Promega, Madison, WI, USA) coding for luciferase DNA and pEGFP-N1 (Clontech, Palo Alto, CA, USA) coding for EGFP DNA. Cy5^TM^ was obtained from Molecular Probe (Mirus, Madison, WI, USA). The Dulbecco’s modified Eagle’s medium (DMEM), 1640 Medium and fetal bovine serum (FBS) were purchased from Invitrogen Corp. MicroBCA protein assay kit was obtained from Pierce (Rockford, IL, USA). Luciferase assay kit was purchased from Promega (Madison, WI, USA). Cell Counting Kit-8 (CCK-8) was purchased from Dojindo Laboratories (Kumamoto, Japan). Naphthalene-1, 4-diyldimethanol was prepared according to the literature [36]. All other reagents used in the synthesis, if not specified, were obtained from J&K Scientific Ltd. (Beijing, China) and used without further purification.

^1^H NMR spectra was obtained on a Bruker AV400 spectrometer (Bruker Corporation, Billerica, MA, USA). CDCl_3_ or D_2_O was used as the solvent and TMS as the internal reference. The molecular weight of polyamine was determined by gel permeation chromatography (GPC) which consisted of a Waters 515 isocratic HPLC pump, a Linear 7.8 × 300 mm column (Waters Corp., Milford, MA, USA), an 18-angle laser scattering instrument (Wyatt Technology Corporation, Santa Barbara, CA, USA), and an OPTILAB DSP interferometric refractometer (Wyatt Technology Corporation, Santa Barbara, CA, USA). A filtered mixture of 0.5 mol/L HAc/NaAc buffer was used as the mobile phase with a flow rate of 1 mL/min. Molecular weights were calculated against poly(ethylene glycol) standards of number average molecular weights ranging from 601 to 106,000.

### 2.2. Synthesis of the Target Polymers

#### 2.2.1. Preparation and Characterization of Linker **L1**–**L3**

Acryloyl chloride (2.00 g, 0.022 mol) in anhydrous dichloromethane (20 mL) was added dropwise to a stirred solution of diol (0.011 mol) and triethylamine (2.24 g, 0.022 mol) in anhydrous dichloromethane (30 mL) under the ice bath. The mixture was stirred overnight at room temperature and then filtered off generated salt, followed by evaporation of the volatile solvent. The residue was purified with silica gel column chromatography (PE:EA = 4:1, v/v) to give **L1**–**L3**.

**L1** colorless solid, yield: 64.34%. ^1^H NMR (CDCl_3_, 400 MHz) δ 6.38 (d, *J* = 17.2 Hz, 2H, –CH=*CH*_2_), 6.15–6.05 (m, 2H, –*CH*=CH_2_), 5.80 (d, *J* = 10.4 Hz, 2H, –CH=*CH*_2_), 4.07 (d, *J* = 7.2 Hz, 2H, –O*CH*_2_–), 3.97 (d, *J* = 6.4 Hz, 2H, –O*CH*_2_–). 1.86–1.76 (m, 2H, –*CH*(CH_2_CH_2_)_2_*CH*–), 1.70–0.96 (m, 8H, –CH(*CH*_2_*CH*_2_)_2_CH–). ^13^C NMR (CDCl_3_, 100 MHz) δ 166.27, 130.54, 128.49, 69.35, 67.19, 37.03, 34.44, 28.79, 25.27. HRMS: calculated for C_14_H_20_O_4_ [M + H]^+^ 253.1434, Found: *m*/*z* = 253.1433.

**L2** white solid, yield: 74.62%. ^1^H NMR (CDCl_3_, 400 MHz) δ 7.37 (s, 4H, –*ArH*), 6.43 (d, 2H, *J* = 17.2 Hz, –CH=*CH*_2_), 6.20–6.10 (m, 2H, –*CH*=CH_2_), 5.85 (d, *J* = 10.4 Hz, 2H, –CH=*CH*_2_), 5.18 (s, 4H, –O*CH*_2_–). ^13^C NMR (CDCl_3_, 100 MHz) δ 165.94, 135.92, 131.22, 128.43, 128.17, 109.99, 65.91. HRMS: calculated for C_14_H_14_O_4_ [M + Na]^+^ 269.0784, Found: *m*/*z* = 269.0785.

**L3** white solid, yield: 55.79%. ^1^H NMR (CDCl_3_, 400 MHz) δ 8.09–8.04 (m, 2H, –*ArH*), 7.58–6.62 (m, 2H, –*ArH*), 7.54 (s, 2H, –*ArH*), 6.43 (d, 2H, *J* = 17.2 Hz, –CH=*CH*_2_), 6.20–6.10 (m, 2H, –*CH*=CH_2_), 5.85 (d, *J* = 10.4 Hz, 2H, –CH=*CH*_2_), 5.65 (s, 4H, –O*CH*_2_–). ^13^C NMR (CDCl_3_, 100 MHz) δ 165.98, 132.55, 131.83, 131.34, 128.13, 126.81, 126.62, 124.25, 64.4. HRMS: calculated for C_18_H_16_O_4_ [M + Na]^+^ 319.0941, Found: *m*/*z* = 319.0943.

#### 2.2.2. Preparation and Characterization of Target Polymer **CyM**, **PhM**, and **NaM**

Polymers were successfully synthesized following modified Michael addition reaction as reported previously [37]. Briefly, Linker **L1**–**L3** (0.75 mmol) and PEI 600 (446.3 mg, 0.75 mmol) were separately dissolved in 2 mL of anhydrous dichloromethane and 2 mL anhydrous methanol, they were mixed in a flask, under a nitrogen atmosphere, the reaction mixtures were heated at 45 °C with constant stirring for 36 h in an oil bath. After the reaction, the solvent was removed under reduced pressure. The mixture of the three crude products was diluted with 3 mL of anhydrous methanol, and then precipitated by the addition of anhydrous diethyl ether. The precipitation was collected and dried in vacuum to get the product as viscous solid. The molecular weights of three target compounds (**CyM**, **PhM**, and **NaM**) were measured by GPC.

**CyM** colorless viscous solid, yield: 59.89%. ^1^H NMR (400 MHz, D_2_O) δ 4.4–3.75 (m, 4H, –O*CH*_2_–), 3.52–3.27 (m, 4H, –COCH_2_*CH*_2_–), 3.13–2.52 (m, 62H, –NH*CH*_2_*CH*_2_–), 2.43 (s, 4H, –CO*CH*_2_CH_2_–), 1.90–0.85 (m, 10H, –*CH*(*CH*_2_*CH*_2_)_2_*CH*–).

**PhM** pale yellow viscous solid, yield: 73.94%. ^1^H NMR (400 MHz, D_2_O) δ 7.38 (s, 4H, –*ArH*), 4.79 (s, 4H, –O*CH*_2_–), 3.38–3.24 (m, 4H, –COCH_2_*CH*_2_–), 3.05–2.50 (m, 72H, –NH*CH*_2_*CH*_2_–), 2.27 (s, 4H, –CO*CH*_2_CH_2_–).

**NaM** white viscous solid, yield: 49.84%. ^1^H NMR (400 MHz, D_2_O) δ 8.16–8.08 (m, 2H, –*ArH*), 7.65–7.59 (m, 2H, –*ArH*), 7.50 (s, 2H, –*ArH*), 5.05 (s, 4H, –O*CH*_2_–), 3.36–3.22 (m, 4H, –COCH_2_*CH*_2_–), 3.01–2.50 (m, 68H, –NH*CH*_2_*CH*_2_–), 2.42 (s, 4H, –CO*CH*_2_CH_2_–).

### 2.3. Agarose Gel Retardation Assay

Polymer/DNA complexes at different mass ratios ranging from 0.2 to 3.2 were prepared by adding an appropriate volume of polymer to 5 μL of pUC19 (0.025 mg/mL). The obtained complex solution was diluted to a total volume of 10 μL, and then the complexes were incubated at 37 °C for 30 min. After addition of 2.5 μL of loading buffer, the complexes were electrophoresed on a 1% (w/v agarose gel containing GelRed™ and with Triseacetate (TAE) running buffer at 150 V for 30 min. DNA was visualized under an ultraviolet lamp using a BioRad Universal Hood II (Berkeley, CA, USA). In the experiments with 10% serum, the complexes solution was obtained by adding PBS buffer and serum, incubating for a certain time. Stability of the polyplex was further analyzed by a polyanion competition assay, appropriate volume of heparin sodium (1.6 mg/mL) was added to the complexes solution and the mixture was incubated for 10 min at room temperature. The samples were run at the same electrophoresis condition as described above. Enzyme stimulated gel electrophoresis experiment was used to investigated the biodegradable properties of the polymers. Complexes were freshly prepared as the method mentioned above. Meanwhile, PEI 25 kDa/DNA complexes were also prepared as a control. After incubation for 30 min, 10 μL esterase from porcine pancreas (10 U/mL) was added into the complexes. The mixture was further incubated at 37 °C for 4 h before electrophoresis.

### 2.4. Ethidium Bromide Displacement Assay

The ability of polymers to condense DNA was studied using EB exclusion assays. Fluorescence spectra were measured at room temperature in air by a HITACHI F-7000 Fluorescence Spectrophotometer (Tokyo, Japan). EB (5 μL, 1 mg/mL) was put into quartz cuvette containing 2.5 mL of 10 mM Hepes solution. After shaking, the fluorescence intensity of EB was measured. Then CT DNA (10 μL, 1 mg/mL) was added to the solution and mixed symmetrically, and the measured fluorescence intensity was the result of the interaction between DNA and EB. Subsequently, the solution of polymer (0.05 mg/mL, 1 μL for each addition) was added to the above solution for further measurement. All the samples were excited at 520 nm and the emission was measured at 600 nm. The pure EB solution and DNA/EB solution without cationic polymer were used as negative and positive controls, respectively. The percent relative fluorescence (%*F*) was determined using the equation %*F* = (*F* − *F*_EB_)/(*F*_0_ − *F*_EB_), wherein *F*_EB_ and *F*_0_ denote the fluorescence intensities of pure EB solution and DNA/EB solution, respectively.

### 2.5. Dynamic Light Scattering (DLS)

The size and zeta potential of complex were evaluated by dynamic light scattering (DLS) at 25 °C by Nano-ZS ZEN 3600 (Malvern Instruments, Malvern, Worcestershire, UK). The polymer/DNA complexes with various w/w ratios were prepared by adding 1 μg of DNA to the appropriate volume of the polymer solution. Then the complex solution was vortexed for 30 s before being incubated at 37 °C for 0.5 h and then diluted up to 1 mL by purity water solution prior to be measured. Data were shown as mean ± standard deviation (SD) based on three independent measurements.

### 2.6. Transmission Electron Microscopy (TEM)

The morphologies of the complexes were observed by TEM (Tecnai G2 F20 S-TWIN, Hillsboro, OR, USA) with an acceleration voltage of 100 kV. 2 μg of pUC19 was added to the appropriate volume of the polymer solution (weight ratio of polymer relative to DNA, w/w = 2:1), then diluted to the total volume of 50 μL. The solution of the complexes was incubated at 37 °C for 0.5 h. 15 min before measurement, the complex solution was diluted with deionized water or water containing 10% fetal bovine serum (FBS) to 1 mL. A drop of DNA/polymer complexes suspension was placed onto the copper grid. After a few minutes, the excess solution was blotted away with filter paper. Then, a drop of 0.5% (w/v) phosphotungstic acid was placed on the above grid. The grid was dried at room temperature at atmospheric pressure for several minutes before observation.

### 2.7. Cell Culture

7702 cells were incubated in 1640 medium containing 10% FBS and 1% antibiotics (penicillin–streptomycin, 10 kU/mL), while HeLa cells were incubated in DMEM medium containing 10% FBS and 1% antibiotics (penicillin–streptomycin, 10 kU/mL) at 37 °C in a humidified atmosphere containing 5% CO_2_.

### 2.8. Amplification and Purification of Plasmid DNA

pGL-3 plasmids used as the luciferase reporter gene were transformed in Escherichia coli JM109. The plasmids were amplified in Luria-Bertani broth at 37 °C overnight. Then the plasmids were purified using an EndoFree Plasmid Kit (TIANGEN, Beijing, China). Then the purified plasmids were dissolved in TE buffer solution and stored at −20 °C. The integrity of the plasmids was confirmed using agarose gel electrophoresis. The purity and concentration of plasmids were determined using ultraviolet (UV) absorbance at 260 and 280 nm.

### 2.9. Cell Viability Assay

Cytotoxicities toward 7702 and HeLa cells were determined using a Cell Counting Kit-8 (CCK-8). Briefly, about 8000 cells per well were seeded in 96-well plates and cultured overnight. A portion of the complexes (100 μL) at various mass ratios of polymer relative to DNA (0.2 μg) were added to the cells in the absence of serum. After 24 h, polymer solutions were removed, and 10 μL of CCK-8 and 90 μL PBS was added to each well for an additional 1 h incubation at 37 °C. Then, the absorbance of each sample was measured using an enzyme-linked immunosorbent assay (ELISA) plate reader (Model 680, Bio-Rad, Berkeley, CA, USA) at a wavelength of 490 nm. The cell viability (%) was obtained according to the manufacturer’s instructions. The untreated cell controls were taken as 100% cell viability. The cell viability of 25 kDa PEI was also performed.

### 2.10. Gene Transfection Efficiency Assay In Vitro

Gene transfection of a series of complexes was investigated in 7702 and HeLa cells. Cells were seeded in 48-well plates (6 × 10^4^ cells/well for 7702, 5 × 10^4^ cells/well for HeLa) and grown to reach 70–80% cell confluence at 37 °C for 24 h in 5% CO_2_. Before transfection, the medium was replaced with a serum-free or a 10% serum-containing culture medium containing polymer/DNA (0.4 μg) complexes at various mass ratios. After 4 h under standard incubator conditions, the medium was replaced with fresh medium containing serum and incubated for another 20 h.

For enhanced green fluorescent protein expression experiment, cells were transfected by complexes containing pEGFP-N1. After 24 h incubation, GFP-expressed cells were observed with an inverted fluorescence microscope (Nikon TS100, Tokyo, Japan). Control transfection was performed in each case using a commercially available transfection reagent PEI 25 kDa based on the standard conditions specified by the manufacture.

For luciferase expression assays, cells were transfected by complexes containing pGL-3. For a typical assay in a 48-well plate, 24 h post transfection as described above, cells were washed with cold PBS and lysed with 60 μL 1 × Lysis reporter buffer (Promega, Madison, WI, USA). The luciferase activity was measured by microplate reader (Imark, Bio-Rad, Berkeley, CA, USA). Protein content of the lysed cell was determined by BCA protein assay (Pierce, Rockford, IL, USA). Gene transfection efficiency was expressed as the relative fluorescence intensity per mg protein (RLU/mg protein). All the experiments were done in triplicate.

### 2.11. Cellular Uptake of Plasmid DNA

The cellular uptake of the polymer/fluorescein labeled-DNA complexes was analyzed by flow cytometry (Accuri C6, BD, Franklin Lakes, NJ, USA). The Label IT Cy5 Labeling Kit was used to label DNA with Cy5 according to the manufacturer’s protocol. Briefly, 7702 and HeLa cells were seeded onto 24-well plates (1.0 × 10^5^ cells/well) and allow to attach and grown for 24 h. Before transfection, the medium was replaced with serum-free or 10% serum culture medium, respectively. Cells were incubated with Cy5 labeled DNA nanoparticles (0.8 μg DNA/well, optimal w/w ratio of each sample) in media for 4 h at 37 °C. Subsequently, the cells were washed with 1 × PBS and harvested with 0.25% Trypsin/EDTA and resuspended in 1 × PBS. Cy5-labeled plasmid DNA uptake was measured in the FL4 channel using the red diode laser (633 nm). Data from 10,000 events were gated using forward and side scatter parameters to exclude cell debris. The flow cytometer was calibrated for each run to obtain a background level of ~1% for control samples (i.e., untreated cells).

To investigate the internalization pathway of the polyplexes, the cellular uptake study was performed at 4 °C or in the presence of various endocytic inhibitors. Briefly, Hela cells were incubated with polyplexes (at optimal weight ratio) at 4 °C for 4 h. Otherwise, Hela cells were preincubated with various endocytic inhibitors including nocodazole (33 µM). cytochalasin D (10 mg/mL) and genistein (200 µM). Following pretreatment for 0.5 h, the inhibitor solutions were replaced by the freshly 10% serum media containing inhibitors at the same concentrations, complexes at the w/w ratio of 1.5 were added directly and further incubated for 4 h. In the study, the groups in the presence of test complexes at 37 °C but without inhibitor treatment were used as controls. Results were represented as percentage uptake level of control cells.

### 2.12. Confocal Laser Scanning Microscopy (CLSM)

HeLa cells were seeded at a density of 2.5 × 10^5^ cells per well in a 35 mm confocal dish (*F* = 15 mm), 24 h prior to transfection. Then, the medium was exchanged with fresh serum-containing medium. Complexes of polycation and Cy5-labeled pGL-3 at the optimal w/w ratio added to each well. After incubation at 37 °C for 4 h, cells were rinsed three times with PBS (pH 7.4), fixed with 4% paraformaldehyde (dissolved with PBS buffer) for 15 min and nuclear staining was done with Hoechst 33342. The CLSM observation was performed using confocal laser scanning microscopy (LSM 780, Zeiss, Jena, Germany) at excitation wavelengths of 405 nm for Hoechst 33342 (blue) and 633 nm for Cy5 (red), respectively.

### 2.13. Statistical Analysis

Statistical analysis was performed using the Student’s *t*-test and differences between the test and control groups were judged to be significant at * *p* < 0.05 and very significant at ** *p* < 0.01 and *** *p* < 0.001.

## 3. Results and Discussion

### 3.1. Preparation and Characterization of Target Polymers

**Scheme 1:**
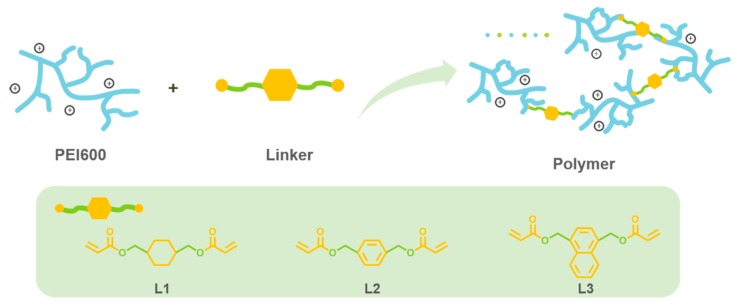
Schematic illustration for the synthetic route of target polymers.

The synthetic route of the title polymers was shown in Scheme 1. The diols with different rigidity was reacted with acryloyl chloride to give the linkers **L1**–**L3**. Subsequently, Michael addition between PEI 600 Da and the linkers would yield the polymers containing ester bonds and linkage with various rigidity. Polymers **CyM**, **PhM**, and **NaM** represent the polymers with cyclohexanyl, phenyl, and naphthyl groups respectively. ^1^H NMR analysis revealed that the actual composition ratio of PEI/linker of the three polymers is close to 1:1, indicating an almost linear structure. GPC was used to measure the molecular weights (*M*_w_) and polydispersity indexes (PDI) of the polymers, which were shown in Table 1. All of the *M*_w_s are much lower than that of PEI 25 kDa, and **NaM** has the lowest *M*_w_, which may be attributed to the steric effect of naphthyl. Besides, we also test two other linkers with phenol moiety, which lack two methylene groups and have higher rigidity compared to **L2**–**L3**. Unfortunately, during their polymerization process, significant color changes were observed (gradually turned into blood red), and products with low viscosity were obtained after purification. We speculate that such linkers might be more liable to be degraded into phenols from the ester hydrolysis under the alkaline condition, leading to oxidation and darkened color.

### 3.2. Interaction with DNA and the Biodegradable Properties

**Figure 1:**
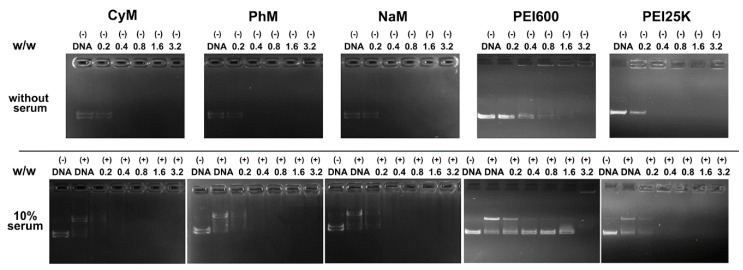
Gel retardation assay of polymer/DNA complexes at different w/w ratio. (−) and (+) represent the condition of serum-free or in the presence of 10% serum, respectively. Naked plasmid DNA was used as control.

**Figure 2:**
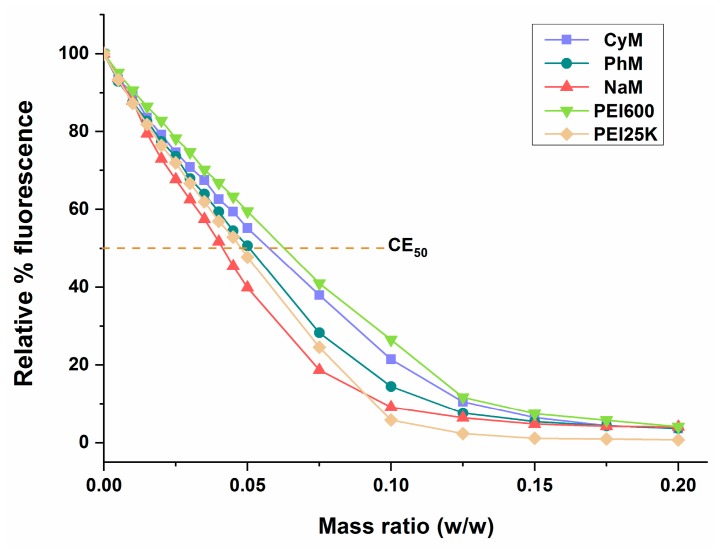
EB exclusion assay aroused by the addition of polymer.

Excellent DNA compression ability is essential for effective gene carrier, and it’s a key factor to avoid DNA degradation by nucleases and promote cellular uptake [38]. DNA condensation ability of the polymers was first characterized by gel retardation assay. As shown in Figure 1, all of the three polymers could fully block the migration of DNA at the polymer/DNA mass ratio (w/w) of 0.4 without or with the presence of serum, and these results were similar to PEI 25 kDa with much higher molecular weight, indicating their good DNA binding ability. On the other hand, the polymerization distinctly enhanced the DNA affinity of PEI 600 Da. Similar results were obtained by the quantitative EB exclusion assay (Figure 2). The relative fluorescence intensity of EB was quenched rapidly with the increase amount of polymer and finally levelled off at ~5% of original intensity. By comparing the CE_50_ values, which are defined as the “charge excess” required to achieve a 50% reduction of fluorescence intensity, we found that **NaM** has the lowest CE_50_ value (even lower than PEI 25 kDa), followed by **PhM**, **CyM**, and PEI 600 Da. This result suggests that the introduction of larger rigid aromatic rings can effectively enhance the DNA binding ability, which might come from the π–π interaction between the aromatic ring and the base pairs.

**Figure 3:**
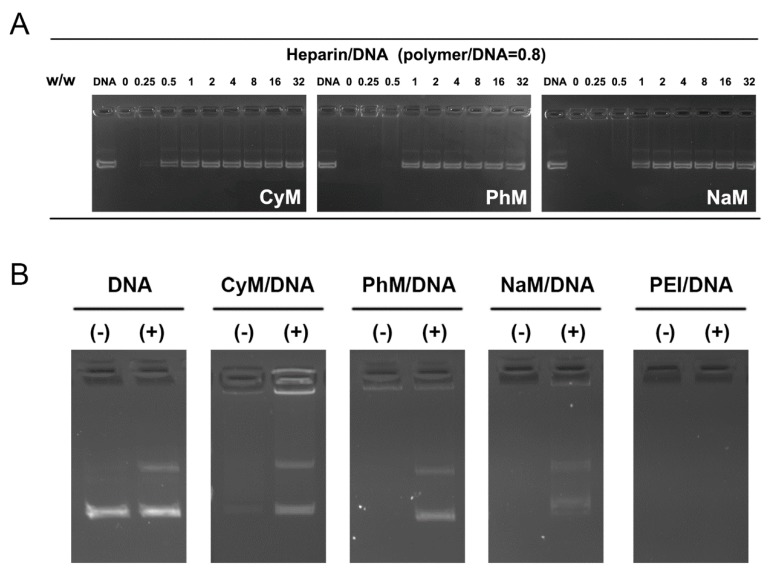
(**A**) The stability of polymer/DNA complexes against heparin. The w/w ratio of polymer/DNA was fixed to 0.8; (**B**) The biodegradable property of the polymers by enzyme stimulated gel electrophoresis experiment. All w/w ratios of polymer/DNA are 0.8. (−): 10 μL of PBS; (+): 10 μL of elastase (10 U/mL).

To further investigate the stability of polymer/DNA complexes (polyplexes), heparin replacement experiment was performed. As shown in Figure 3A, for **CyM**/DNA polyplex, DNA release was found from the heparin/DNA weight ratio of 0.25, and obvious DNA band could be observed at the ratio of 0.5. Meanwhile, for the aromatic ring-contained polymers **PhM** and **NaM**, more amount of heparin (w/w = 1) was needed to release DNA, indicating that the aromatic ring help to form the polyplex with higher stability. In addition, the degradability of the polymers was also studied by enzyme stimulated gel electrophoresis experiment. Elastase, which can catalyze the hydrolysis of ester bonds, was added to the polyplexes. Results in Figure 3B show that after incubation at 37 °C for 4 h, the DNA band reappeared in the experiment groups involving all of the title polymer/DNA complexes. On the contrary, no DNA release was found in the PEI 25 kDa control group. These results demonstrate that the synthesized polymers have degradable potential in cytoplasm where the hydrolase existed, facilitating DNA release and subsequent gene transfection.

### 3.3. Characterization of the Polyplexes

**Figure 4:**
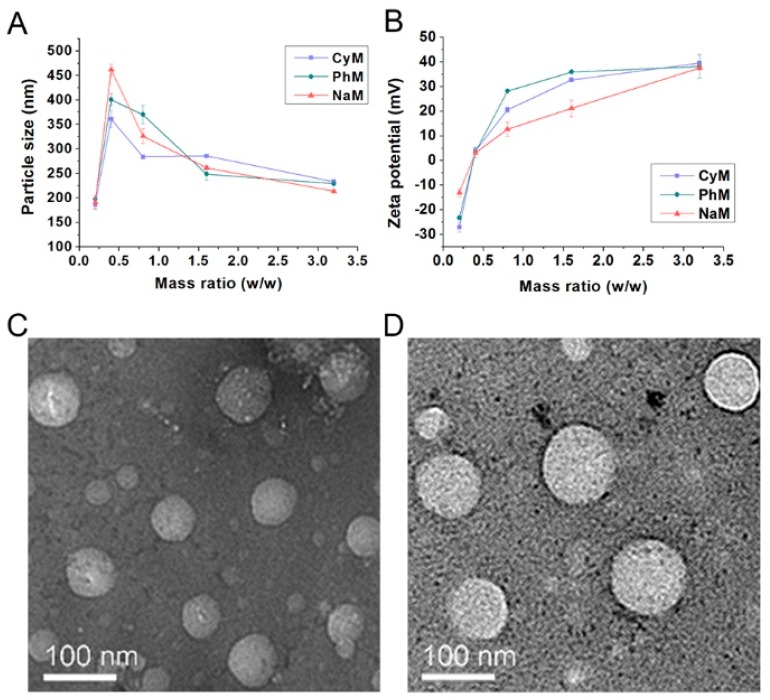
Characterization of the polyplexes. Mean particle sizes (**A**) and zeta potentials (**B**) of the polyplexes at various mass ratios measured by DLS. TEM images of **NaM**/DNA polyplex (w/w = 4) in water (**C**) and in water with 10% serum (**D**).

Generally, appropriate particle size and surface potential are necessary for efficient cellular uptake [39,40]. Dynamic light scattering experiment was carried out to measure these physical properties of the polyplexes at different mass ratio. As shown in Figure 4A,B, at the w/w of 0.4, the polyplexes gave maximum size, and this could be due to their electric neutrality at such w/w, resulting in less repulsion between the particles and more liability to aggregate. With the increase of mass ratio, the particle size decreased and became steady, suggesting that the polymers could condense DNA into complexes with diameters of ~220 nm. Meanwhile, the zeta potential increased with the raise of w/w, and finally reached ~+35 mV, exhibiting obvious positive charge. In addition, the morphology of **NaM**/DNA complex was imaged by TEM. Figure 4C illustrated that **NaM** condensed DNA into uniform spherical nanoparticles with the average diameter of ~75 nm, which was much smaller than that obtained by DLS. This inconsistence could be attributed to the different test conditions in these assays. DLS provide a hydrodynamic size of the particles, which can further aggregate to form larger particles. While in TEM measurements, the particles are obtained by adsorption on the surface of carbon coated Cu meshes [41]. With the presence of 10% serum, the shape of the polyplex was seldom changed (Figure 4D), and the diameter of the particles only slightly increased to 85 nm, indicating their good serum tolerance.

### 3.4. Cytotoxicity

**Figure 5:**
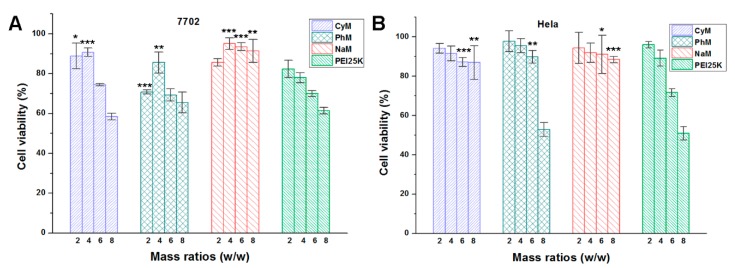
Cytotoxicity of the polyplexes at various w/w ratios in 7702 (**A**) and HeLa (**B**) cells. * *p* < 0.05; ** *p* < 0.01; *** *p* < 0.001 vs. PEI complexes.

For most of the cationic gene vectors, the transfection efficiency is always closely linked with cytotoxicity [40,42]. Therefore, balancing transfection efficiency and cytotoxicity is of great importance for designing safety and efficiency delivery vectors. The toxicity of the three polyplexes at various w/w ratios was assessed in both 7702 and HeLa cells by CCK-8 assay. As shown in Figure 5, compared to PEI 25 kDa, under the same w/w ratio, the synthesized materials induced similar or higher cell viability. **NaM** showed very low cytotoxicity in both cell lines, even at high w/w ratio. **CyM** also led to high viability, especially in HeLa cells at higher w/w ratio. Such lower toxicity may be derived from the joint contribution of the lower molecular weight and the biodegradable ester bond in the polymer backbone.

### 3.5. In Vitro Gene Transfection

**Figure 6:**
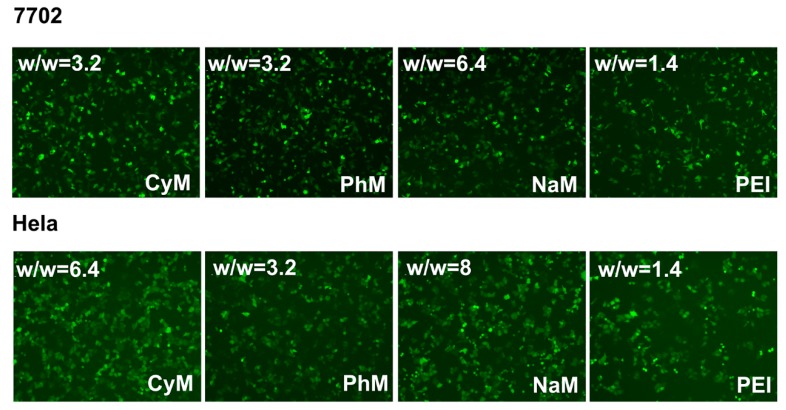
GFP expression in 7702 and HeLa cells induced by the polyplexes at the optimal mass ratio. Images were taken by inverted fluorescence microscope.

To assess the gene delivery capacity of these polymeric vectors, polyplexes were first tested in 7702 and HeLa cells by enhanced green fluorescent protein (eGFP) expression experiment. The polyplex formed from PEI 25 kDa was used as control. For cationic polymers, higher molecular weight always leads to better transfection [16]. From the fluorescent images (Figure 6), it could be found that although the molecular weights of the title polymers were only half of PEI 25 kDa, they could induce the green fluorescence with higher density than PEI did, indicating their higher transfection efficiency. It is worth mentioning that linkers containing aromatic rings and diol glycidyl ether were also designed aiming to obtain polymers by ring-opening polymerization. Compared to the polymers reported here, those materials exhibited poor to moderate transfection efficiency together with significant cytotoxicity (data not shown). This may result from the combined effect of their non-degradability and polycationic property.

**Figure 7:**
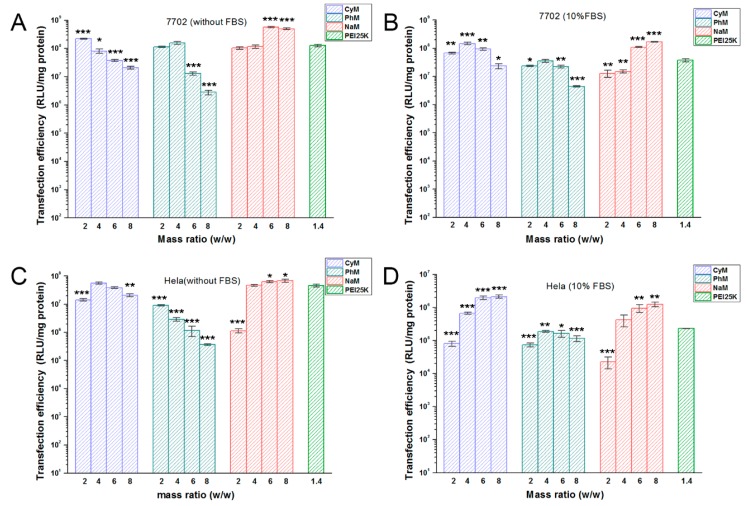
Luciferase expression of mediated by the polyplexes at various w/w in 7702 (**A**: without FBS, **B**: with 10% FBS) and HeLa cells (**C**: without FBS, **D**: with 10% FBS). * *p* < 0.05; ** *p* < 0.01; *** *p* < 0.001 vs. PEI complexes.

Subsequently, the transfection was quantitatively investigated by using luciferase reporter gene. Results revealed that all materials exhibited comparable or superior transfection efficiency compared to PEI. Among the three polymers, **NaM** with larger aromatic rings gave apparently better transfection than **PhM**, and about 4.5- and 1.5-times higher efficiencies than PEI were obtianed in 7702 (Figure 7A) and Hela cells (Figure 7C), respectively. The transfection was also performed in the presence of serum. Usually, anionic species in serum might interact with the cationic vectors and negatively affect the stability of the polyplex, leading to decreased transfection efficiency [43]. As shown in Figure 7B,D, compared to the much decreased efficiency of PEI, our materials showed better serum tolerance, which could be attributed to their higher DNA binding ability and higher stability of the formed polyplex. It is worth mentioning that **CyM** could also give higher transfection efficiency than PEI. Combined with the lower cellular uptake (especially the fluorescent intensity result with the presence of serum) mediated by **CyM**, we speculate that its good transfection efficiency might come from the better DNA release ability (Figure 3), leading to better intracellular delivery.

### 3.6. Cellular Uptake

**Figure 8:**
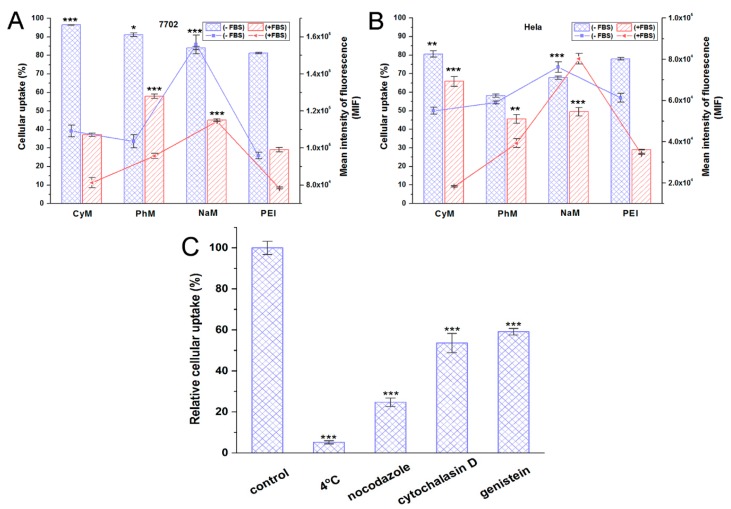
Cellular uptake of complexes with 10% serum at optimal w/w ratio in 7702 (**A**) and HeLa (**B**) cells quantified by flow cytometry. Columns: percentage of Cy5-positive cells; Dots and lines: fluorescence intensity of internalized Cy5-labeled DNA. * *p* < 0.05; ** *p* < 0.01; *** *p* < 0.001 vs. the mean intensity of fluoresscence of PEI complexes; (**C**) Cellular uptake level of **NaM**/DNA polyplexes (w/w = 8) in HeLa cells in the presence of various endocytic inhibitors. * *p* < 0.05; ** *p* < 0.01; *** *p* < 0.001 vs. control.

To study the effect of aromatic ring bridge ligand on cellular uptake, 7702 and HeLa cells were incubated with the Cy5-labeled polyplexes at their optimal w/w and then measured by flow cytometry, and the results are shown in Figure 8. All materials could give high percentage of Cy5-positive cells in serum-free condition. Compared the three polymers, **PhM** led to a relatively lower uptake percentage, which might the reason of its lower transfection efficiency (Figure 7). Serum indeed had a negatively effect on both cellular uptake percentage and the internalized fluorescence intensity. However, it could be seen that for the polymers with aromatic ring bridge (**NaM** and **PhM**), the negative effect was less. On the contrary, **CyM**-mediated cellular uptake suffered a large decrease, which was due to its lower DNA binding and protecting ability. For **NaM** in HeLa cells, even higher Cy5 fluorescent intensity was obtained in the presence of serum (Figure 8B). The results also demonstrate that aromatic rings in the polymer structure may facilitate their interaction with DNA and the formation of more stable polyplex. On the other hand, although **NaM** did not give the highest cellular uptake percentage, it induced the highest internalized fluorescence (especially in the presence of serum), suggesting that **NaM**/DNA polyplex experienced the most efficient cellular uptake. This may give us some clues for the rational design of gene vectors that can effectively protect and deliver DNA into cells.

To further investigate the internalization pathway of the polyplex, **NaM** was selected to study cellular uptake level at low temperature or in the presence of various endocytic inhibitors. As shown in Figure 8C, the cellular uptake of **NaM**/DNA polyplexes were blocked ~95% at 4 °C, indicating that polyplexes enter the cells mainly via energy-dependent process. Nocodazole, which can inhibit the pathway of microtubule-mediated endocytosis, causes a remarkable reduction of cellular uptake (more than 70%). In addition, as the inhibitors of macropinocytosis and caveolae respectively, cytochalasin D and genitstein also exhibited an inhibitory effect. Such results indicate that the polyplexes enter cells mainly via microtubule pathway. Meanwhile, macropinocytosis and caveolae pathways are also accepted during cellular uptake.

**Figure 9:**
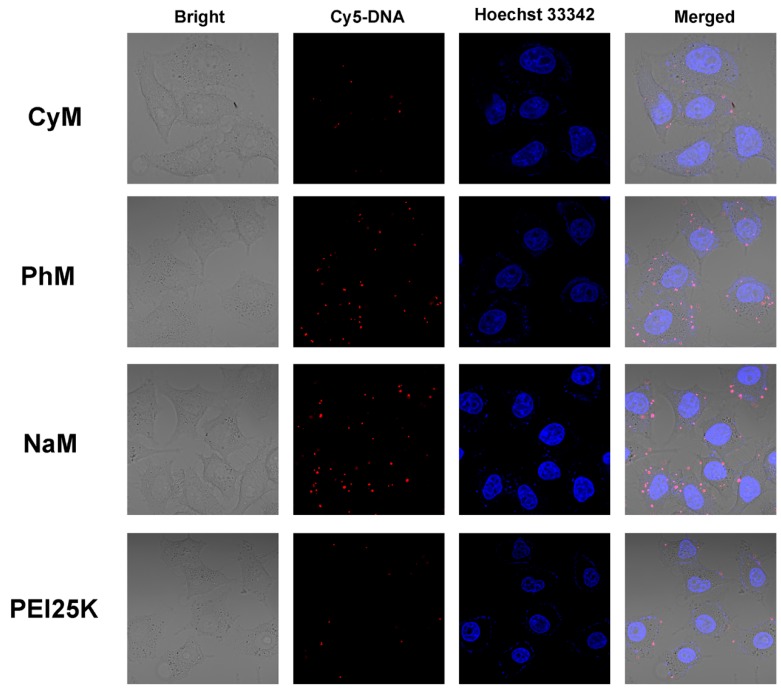
CLSM images of HeLa cells transfected with Cy5-labeled DNA by the complexes at the optimal w/w ratio after 4 h.

Confocal laser scanning microscopy (CLSM) was also performed to visualize the internalization of Cy5-labeled DNA (red) delivered by the vectors at their optimal w/w ratio in HeLa cells. As shown in Figure 9, after 4 h incubation with polyplexes under the condition of 10% FBS, clear red signal of Cy5-labled DNA appeared in the cells. With the increase of rigidity of polymer backbone, more red signals could be observed. **NaM** with larger aromatic rings could induce the red fluorescence with the highest density, while **CyM** only led to few fluorescent signals in the presence of serum, which was consistent with the results of flow cytometry.

## 4. Conclusions

In summary, we designed and prepared a series of biodegradable polymeric gene carriers based on LMW PEI 600 Da by ester linkages with different rigidity. The synthesized materials could effectively condense DNA to spherical particles with diameters of ~220 nm and a positive surface charge of ~+35 mV. Gel electrophoresis results confirmed their DNA binding ability and biodegradability to release DNA cargo. These materials have lower molecular weights compared to PEI 25 kDa, but exhibited higher transfection efficiency, especially in the presence of serum. The rigid aromatic rings in the polymer structure enhanced their DNA affinity and improved the stability and cellular uptake of the polyplexes, which was confirmed by various experiments including flow cytometry and CLSM. In conclusion, this strategy for the construction of non-viral gene vectors may be applied as an efficient and promising method for gene delivery.

## Figures and Tables

**Table 1:** *M*_w_ and PDI of polymers measured by GPC.

Polymers	*M*_w_	PDI
**CyM**	10,555	2.24
**PhM**	11,940	2.50
**NaM**	9075	1.90
